# Copy number variation in African Americans

**DOI:** 10.1186/1471-2156-10-15

**Published:** 2009-03-24

**Authors:** Joseph P McElroy, Matthew R Nelson, Stacy J Caillier, Jorge R Oksenberg

**Affiliations:** 1 Department of Neurology, University of California, San Francisco, CA, USA; 2 GlaxoSmithKline, Research Triangle Park, NC, 27709, USA

## Abstract

**Background:**

Copy number variants (CNVs) have been identified in several studies to be associated with complex diseases. It is important, therefore, to understand the distribution of CNVs within and among populations. This study is the first report of a CNV map in African Americans.

**Results:**

Employing a SNP platform with greater than 500,000 SNPs, a first-generation CNV map of the African American genome was generated using DNA from 385 healthy African American individuals, and compared to a sample of 435 healthy White individuals. A total of 1362 CNVs were identified within African Americans, which included two CNV regions that were significantly different in frequency between African Americans and Whites (17q21 and 15q11). In addition, a duplication was identified in 74% of DNAs derived from cell lines that was not present in any of the whole blood derived DNAs.

**Conclusion:**

The Affymetrix 500 K array provides reliable CNV mapping information. However, using cell lines as a source of DNA may introduce artifacts. The duplication identified in high frequency in Whites and low frequency in African Americans on chromosome 17q21 reflects haplotype specific frequency differences between ancestral groups. The generation of the CNV map will be a valuable tool for identifying disease associated CNVs in African Americans.

## Background

Duplications or deletions of genomic segments generate copy number variants (CNVs) that can range is size from one thousand to several million base pairs, and may affect one or more genes. More nucleotides appear to be affected by CNVs than by single nucleotide polymorphisms (SNPs) [1]. Current annotated CNVs cover about 28.8% of the genome, and, to date, over 5600 non-overlapping human CNV loci have been identified ; Database of Genomic Variants) [2]. CNVs are a major source of human genetic diversity, and have been shown to influence rare genomic disorders [3] as well as complex traits and diseases [4].

In addressing the role of CNVs in disease, it is important to understand their distribution in the population at large [5]. Several studies have attempted to characterize CNVs in the general population using data from the International HapMap Consortium [1,6-8], and other reference groups [2,5,9-11], and have confirmed that CNVs are widespread throughout the genome but show a broad range in population frequencies. However, as of the preparation of this manuscript, no reported studies have surveyed CNVs in African Americans. The objectives of the current study are to use genome-wide SNP array data to generate a CNV map of the African American genome and to describe differences between African and European Americans.

## Methods

### Experimental Populations

DNAs of 435 healthy African Americans and 435 healthy individuals of European descent (hereafter referred to as Whites) were available for analysis. High molecular weight DNA was extracted from freshly isolated peripheral blood lymphocytes using a standard desalting procedure. Quality and quantity of each genomic DNA sample was evaluated by fluorometry (Molecular Devices Spectra Max). One hundred forty of the African American DNA samples were derived from lymphoblastoid cell lines, all of which were from females, and all other DNA was isolated from whole blood. Epstein Bar virus (EBV)-transformed lymphoblastoid lines were generated from freshly isolated peripheral blood lymphocytes. Cells were washed and resuspended in complete Iscoves modified Dulbeccos culture media supplemented with 10% v/v fetal bovine serum, antibiotics, and virus. The ATCC B95-8 EBV-infected marmoset cell line was used as the source for virus stocks. The UCSF institutional review board approved this study and all participants gave written informed consent.

African American individuals were recruited from 28 US States, the mean age at sample acquisition was 45 years, and the population displayed a wide range of admixture [12]. African American ancestry was self reported, but European ancestry was documented in the majority of individuals based on genotyping of 186 SNPs highly informative for African versus European ancestry as previously described [13]. Global estimation of European ancestry using these markers indicated 23 ± 15% European ancestry [14]. White individuals originated from 8 different regions: Australia (n = 11), East Europe (n = 22), North Africa (n = 1), North America (n = 29), North Europe (n = 93), South America (n = 1), South Europe (n = 71), and West Europe (n = 207). Females constituted 64% and 51% of the African American and White populations, respectively. All individuals were assayed on the Affymetrix GeneChip^® ^Human Mapping 500 K Array Set. Quality control filtering and SNP frequencies are reported elsewhere [12].

### Data Analysis

Fifty randomly chosen African American females with DNA derived from whole blood were used as references for calculating the normalized total intensity measures for each SNP (log-R ratios) for all of the remaining individuals. The reference individuals were excluded from further analysis, resulting in 385 African American and 435 White test individuals. Using only female references allows the estimation of X chromosome CNVs in female test individuals. Raw copy number files (".cnchp" files) were generated using the CNAT4.0.1 algorithm in the Affymetrix^® ^Genotyping Consol™ 2.1 with default settings. The ".cnchp" files from both the African American and White individuals were read into the Nexus 3.0 copy number analysis program (BioDiscovery, Inc.) and copy number variable regions were called using BioDiscovery's rank segmentation algorithm [15] with default settings for the Affymetrix 500 K assay which requires at least one probe per segment. CNV frequencies and between group frequency differences were estimated using Nexus. Fisher's Exact test was used to determine the significance of the frequency differences and False Discovery Rate (FDR) [16] was used to correct for multiple comparisons.

### qPCR

CNVs of interest were validated using region-specific TaqMan assays. An internal positive control gene (β-globin, *HBB*) was included in each assay to determine copy number and to confirm that the reaction amplified successfully [see Additional file 1]. Threshold cycle (Ct) values were generated from a pre-established threshold and Δ_Ct _values were estimated from the difference of the control gene and the CNV test region. The Δ_Ct _values were then treated as a quantitative trait and standard analysis of variance was utilized to test the association of the SNP-determined CNV status with the Δ_Ct _for that region.

## Results

DNAs from 385 healthy African American and 435 healthy White individuals were scanned using the Affymetrix GeneChip^® ^Human Mapping 500 K Array Set to identify CNVs. A single African American individual's DNA was plated twice, and is used as a comparison for consistency for CNV calls using the Affymetrix 500 K platform. Based on the log-R ratios, evidence for four identical CNVs was present in both samples, although a single deletion on chromosome 21 identified in one sample was just below the call threshold in the other sample [see Additional file 2]. The consistency of the results indicates the reproducibility of the experiment, albeit only in a single sample.

Based on the distribution of the number of CNV calls per individual [see Additional file 3], 28 individuals were identified as outliers (due to high numbers of CNV calls) and removed from the analysis to reduce the probability of CNV calls that were a result of assay performance rather than the presence of true CNVs. In addition, all CNVs on the X chromosome identified in males were removed, since all males have deletions of a single copy of the X chromosome when compared to female references.

Autosomal CNVs were contrasted between African American males and females to establish a conservative threshold for the largest CNV frequency differences expected under the null hypothesis, since true autosomal differences between males and females are not expected. The largest frequency difference for any autosomal CNV between African American males and females was 6.6%. Performing the same experiment in Whites yielded a largest autosomal CNV frequency difference between males and females of 5%. None of the CNV regions in either group with a frequency difference of 5% or greater between males and females harbored genes that were obvious candidates for sexual dimorphism. Since the largest frequency difference observed between males and females was 6.6%, a conservative threshold of 10% will be used in combination with the Fisher's Exact test FDR corrected p-values to declare true differences for further comparisons.

While all of the DNA samples for the White individuals were isolated from whole blood, 140 of the African American DNAs were isolated from lymphoblastoid cell lines. DNA derived from cell lines may have CNVs that result from the establishment of the lines [17]. Any high frequency CNVs in the African American group that arose from the process of creating cell lines need to be identified and removed from the comparison between African and Whites. Considering only African American subjects, three regions showed a significant difference greater than 10%: chromosome 14 (21,811,993 – 21,836,082) (duplication in 74% of cell line DNAs; FDRp < 0.001), chromosome 14 (105,619,582 – 106,173,672) (deletion in 10.7% of cell line DNAs; FDRp < 0.001), and chromosome 17 (41,592,674 – 41,597,102) (duplication in 11.03% of cell line DNAs; FDRp < 0.002). CNVs in cell lines in these regions will not be considered in further comparisons between African American and White CNVs.

### CNV Detection

**Figure 1:**
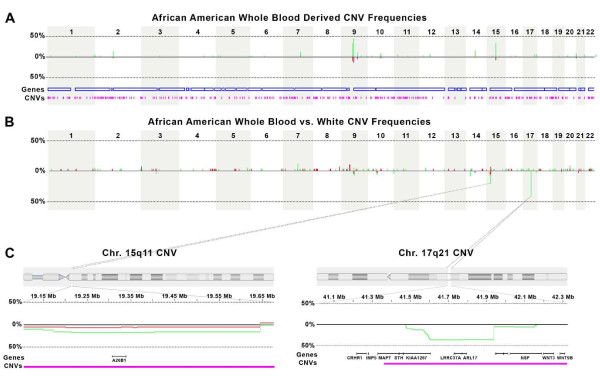
**Distribution of CNVs and differences in CNV frequencies**. **A**. Genomic distribution of CNV frequencies in the African American population. Green bars above the central line indicate duplications at those genomic positions and red bars below the central line indicate deletions at those genomic positions. The height of the bars indicate the frequency of the CNV at a given genomic position in the population. **B**. Genomic distribution of CNV frequency differences between whole blood derived African American and White DNAs. A green or red bar above the central line indicates a higher frequency of duplications or deletions, respectively, in the whole blood DNA at the given genomic position, and the height of the bar indicates the magnitude of those frequency differences. Likewise, bars below the central line indicate higher frequencies in cell line DNAs. **C**. Close up of differences of CNV frequencies between African Americans and Whites for the chr. 15q11 and 17q21 regions. Green line below the central line indicates and increased frequency of duplication in Whites, and above in African Americans. Red line below the central line indicates and increased frequency of deletion in African Americans, and above in Whites. The lines below the chromosomal graph indicate the gene locations and locations of CNVs in the Database of Genomic Variants (pink bar).

In the 384 African Americans, a total of 1362 copy number events were identified, with a mean of 3.5 CNVs per individual vs. the reference panel (results for DNAs isolated from whole blood are shown in Figure 1A). A total of 1972 copy number events were identified in Whites, resulting in a mean of 4.8 CNVs per individual. The higher CNV frequency in Whites (Wilcoxon rank-sum test p < 0.0001) was not surprising since the reference group consists of African American genomes. The average size of duplications and deletions in African Americans were 827 kb and 703 kb, respectively (Wilcoxon rank-sum test p = 0.031), and the average size of duplications and deletions in Whites were 671 kb and 708 kb, respectively (Wilcoxon rank-sum test p < 0.0001). Counting each CNV region as a different feature unless both borders were identical (border-matched), 1068 CNV regions were identified across all individuals. For these border-matched CNV regions, 412 were unique to African Americans, 580 were unique to Whites, and 76 were common between the two populations. Excluding CNVs that occurred only in a single individual, 27 were unique to African Americans, 71 were unique to Whites, and 76 were common [see Additional file 4]. The highest frequency CNV regions identified in African Americans were duplications on chromosomes 9 and 15 (15: 30.83%; 19,643,165 – 19,978,503). The duplication on chromosome 9 was divided into three sections (41,217,099 – 46,875,500) separated by segments without SNPs (the two flanking segments were tagged only with single SNP because there were no other SNPs in the immediate vicinity), and therefore is likely a single CNV. Across this region, the highest frequency of the duplication in the African American population was 48.7%. One of the chromosome 9 single SNP tagged-CNVs is in a region not identified in the Database of Genomic Variants. In addition, a CNV region on chromosome 5 (162,208,673 – 162,463,912) was identified in two African American individuals that was not in the Database of Genomic Variants. All other African American CNVs identified in two or more individuals overlapped at least partially with regions denoted as CNVs in the Database of Genomic Variants [2].

Two regions were markedly different between African Americans and Whites, excluding cell line regions (Figure 1B and 1C). A duplicated region was identified on chromosome 17 (41,600,030 – 41,932,225) that had a frequency of 45.1% in Whites and 8.03% in African Americans (FDRp < 0.001). Two genes are annotated in this region: leucine rich repeat containing 37A (*LRRC37A*) and ADP-ribosylation factor-like 17 (*ARL17*). Another duplicated region was identified on chromosome 15 (19,212,556 – 19,400,776) with a frequency of 21.24% in African Americans and 40.69% in Whites (FDRp < 0.001). The gene ANKRD26-like family B, member 1 (*A26B1*) is in this region. None of the aforementioned genes appear to have a readily identifiable biological association with ethnic differences. All other CNV features had a difference of <10% between African Americans and Whites.

Extreme copy events (homozygous deletions and >1 copy gains) were also analyzed independently from the previous analysis for differences between the two populations. In total, 75 extreme copy events were identified in African Americans (70 gains and 5 losses) and 176 extreme copy events were identified in Whites (171 gains and 5 losses). None of the frequencies of the extreme copy event regions were greater than 10% different between African Americans and Whites, but a single region was significantly different (p < 0.05) after FDR correction on chromosome 15 (18,427,103 – 19,643,166). This multiple copy gain in this region had a maximum frequency of 0.013 in African Americans and 0.086 in Whites. The two genes located in this region (coxsackie virus and adenovirus receptor pseudogene 2 [*CXADRP2*] and POTE ankyrin domain family member B [*POTEB*]) do not have an immediately apparent functional association with ethnicity.

In addition to the cell line associated CNV regions identified in the current study, copy number variations of chromosome 2 (88,876,198–89,912,849; 0.093 frequency in cell line derived DNAs and 0.024 frequency in whole blood derived DNAs), and deletions of chromosome 22 (20,905,109–21,439,970; 0.029 frequency in cell line derived DNAs and 0 frequency in whole blood derived DNAs) have previously been shown to be artifacts of transformation or somatic recombination of immunoglobulin genes ([17] and [18], respectively). Although these regions did not meet the criteria (FDR significant and >10% frequency difference) to be identified as associated with the generation of cell lines in the current study, they will be excluded from the data submitted to the Database of Genomic Variants, as will three regions labeled as copy number variant based on the data from a single SNP (because of sparse SNP spacing in these regions). All other CNVs identified the current study have been submitted to the Database of Genomic Variants [2].

### qPCR

**Figure 2:**
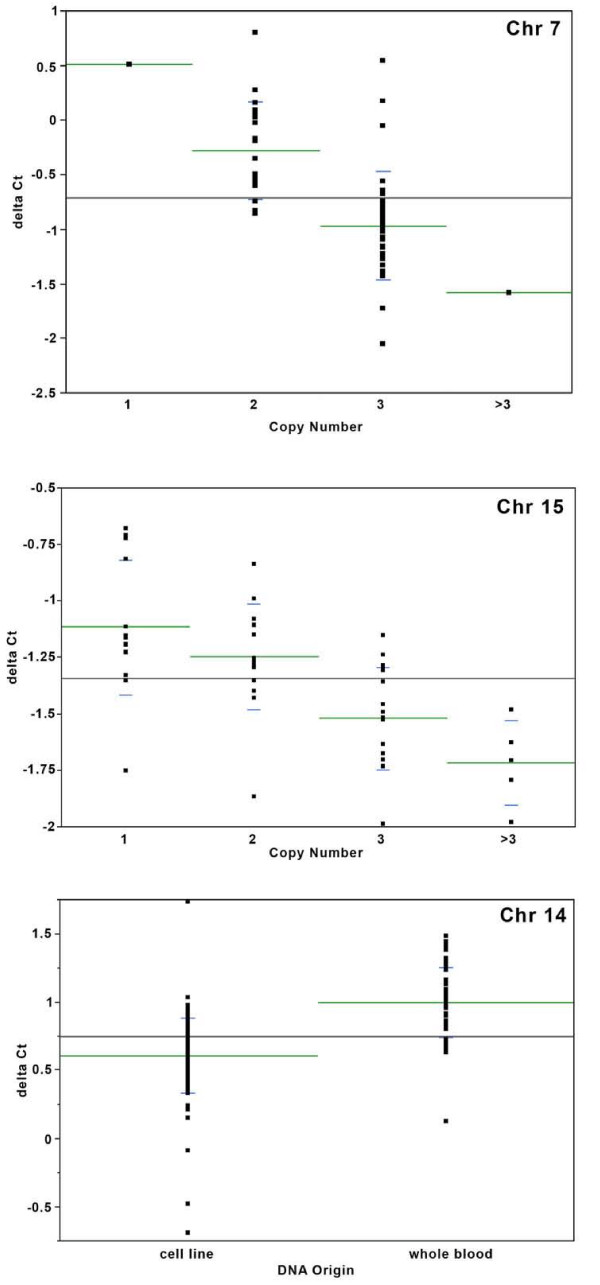
**Delta ct distributions for ANOVA of three CNVs validated by qPCR**. P-values for ANOVAs were p = 0.00001 (Chr. 7q11), p = 0.0001 (15q11), and p = 1.2e^-12 ^(14q11). Lower delta ct values indicate higher copies. Green lines indicate means, blue lines indicate standard deviations. Primer sequences for the three CNV regions: chromosome 7 forward-5' TGC CAC TTG CGT TCT T 3', reverse-5' CTT GGG CCA CGT CAT T 3'; chromosome 14 forward-5' CAC TGG CAT TTG GTA TCG T 3', reverse-5' CCC AAA GTG AAA CGT ATT 3'; chromosome 15 forward-5' ATG CCA CAT ATT CTT ACT CAT 3', reverse-5' CCA CAC TCC ACC CTC AA 3'.

In order to assess the robustness of the Affymetrix 500 K array to identify CNVs, qPCR was performed in a subset of the African American samples on three representative CNV regions, selected based on the frequency differences between the two populations (chromosomes 15 and 7) and the differences between cell lines and blood (chromosome 14). Although it did not reach the 10% difference threshold, the chromosome 7 region (76,052,765 – 76,371,008) (FDRp < 0.001) was chosen over the chromosome 17 region because there were more African Americans with the chromosome 7 CNV. In addition, both cell line and blood-derived DNAs were included for ten individuals with the chromosome 14 duplication. Figure 2 depicts the graphs of the Δ_Ct _values for each of the ANOVA comparisons. In every case, the mean of the Δ_Ct _was in the expected direction, confirming the presence of the CNVs, i.e., the higher the number of copies indicated by the SNP analysis for a region, the lower the mean Δ_Ct _value for that group. In addition, for each of the ten individuals in which cell line and blood derived DNAs were included, the cell line DNAs had a lower Δ_Ct _than the whole blood DNAs for the chromosome 14 duplication, indicating that indeed there were more copies of the region in the cell line DNAs [see Additional file 5].

## Discussion

In the current study, a CNV map was generated using DNA from a population of 385 African Americans using 50 randomly chosen female African Americans as a reference. A total of 1362 CNV events were identified in the population. In addition, CNVs were identified in a population of 435 White individuals using the same 50 African American females as a reference. The same reference population was used so that the CNV distributions of the two populations would be directly comparable. Two regions of the genome exhibited large CNV frequency differences between the two populations, one on chromosome 15 and another on chromosome 17. No genes in these regions had obvious roles in ethnic differences.

A total of 140 of the African American DNAs were derived from cell lines. The process of creating the cell lines generated a duplication on chromosome 14 in 74% of the cell line-derived DNAs. Although this region is listed as copy number variant in the Database of Genomic Variants, none of the DNAs derived from whole blood was identified as having this duplication. Apparently, either transfection with the EBV virus or the growing out of the cells caused this duplication event. The EBV virus may have integrated into this site, disrupting the organization of the region and resulting in the duplication. However, Jeon and colleagues did not identify a CNV in this region resulting from EBV transformation of B-cells from Korean subjects, and the 1p36.33 copy number increase identified in cell lines by Jeon et. al was only found in a cell line from a single individual in the current study [17]. Simon-Sanchez and colleagues also did not identify this CNV when comparing DNA from EBV transformed cell lines to blood derived DNAs in a cohort of North American Whites [19]. Another possibility is that the integration of the EBV DNA into another site of the genome may facilitate duplication at this site. Finally, a gene in this region may facilitate the process of expansion or survival of the cell line, and therefore cells with this duplication may have been selected for in the culturing and growing process. However, there are no annotated genes in the region of the duplication. Currently, it is unknown if the duplication is an ethnic, experimental, or EBV strain specific phenomenon, and the determination of these specifics is under investigation.

A duplication on chromosome 17 (41,600,030 – 41,932,225) was identified in both African Americans and Whites in the current study. This duplication is in the same location as a segmental duplication flanking a mental retardation associated deletion identified in another study [20]. Segmental duplications have been shown to be catalysts for chromosomal rearrangement [10]. Two major haplotypes (H1 and H2) are present in this region of the human genome, and the ancestral haplotype (H2), which is more prone to duplications, is found mostly in people of European descent (see [21] for discussion of 17q21.31). Most Africans have the H1 haplotype, which may explain the large frequency difference of the duplication in this genomic region between African Americans and Whites. Since the present study found that the duplication was present in 45% of Whites and only 8% of African Americans, it will be of interest to assess if the severe neurological phenotype resulting from the deletion in the 17 region is more prevalent in Whites than in Africans or African Americans. *CRHR1 *(corticotrophin releasing hormone receptor 1) and *MAPT *(microtubule-associated protein tau) are two of the six genes within the region deleted as a result of the segmental duplication. These genes are both associated with many neurological disorders. Since it is close to the genes, it is important to determine whether the duplication has an effect on the expression of these genes, which could produce a neurological phenotype.

## Conclusion

As of the preparation of this manuscript, there are no other reports of the production of a CNV map in African Americans. The creation of this map is an important first step in determining the presence CNV admixture in African Americans. Since many studies are now identifying CNVs as underlying causes in disease subsets, the African American CNV map will also be important for identifying cross-ethnic and ethnic-specific disease associated CNVs.

## Authors' contributions

JM and JO conceived of the study and participated in its design and coordination. JM performed all statistical analyses and drafted the manuscript. MN carried out the Affymetrix SNP assays. SC carried out the qPCR assays. All authors read, edited, and approved the final manuscript.

## Supplementary Material

Additional file 1 **qPCR supplemental methods.** Additional information on the qPCR methods used in this manuscript. Click here for file

Additional file 2 **Replication of CNV analysis in a single individual.** A. CNV calls in replicate individual. A green bar above the line for an individual indicates a duplication at that region in the genome, and a red bar below indicates a deletion at that region in the genome. B. Log R ratios for SNPs on chromosome 21. Arrows indicate the region designated as deleted in the first replicate, but not in the second replicate. Click here for file

Additional file 3 **Distribution of the numbers of CNV calls per individual before (A) and after (B) removing outliers.** The upper panels of each figure are box and whiskers plots of the data. Each box indicates the interquartile range, each line across the boxes indicates the median, the diamonds indicate the means and 95% confidence intervals, and the whiskers indicate the upper quartile + 1.5 × interquartile range (right side of boxes) and the lower quartile – 1.5 × interquartile range (left side of boxes). Click here for file

Additional file 4 **CNVs identified in more than a single individual.** Location, types of CNV events, and within population frequencies of CNVs identified in two or more individuals. Events (relative to reference population): HDel = two copies decrease, Del = one copy decrease, Dup = one copy increase, HDup = two or more copy increase. Click here for file

Additional file 5 **qPCR of chr. 14 replicates (red cell line, blue whole blood).** Bar chart of delta Cts for ten individuals with both whole blood and cell line derived DNAs for the chromosome 14q11 cell line associated CNV. Red bars are cell line DNAs, blue bars are whole blood DNAs. In every individual, the cell line DNAs have a lower delta Ct. Primer sequence for chromosome 14 qPCR: forward-5' CAC TGG CAT TTG GTA TCG T 3', reverse-5' CCC AAA GTG AAA CGT ATT 3'. Click here for file
