# Supplementary material for: Copy number variation in African Americans
Source: BMC Genet. 2009 Mar 24;10:15. doi: 10.1186/1471-2156-10-15 (PMC2674062; doi:10.1186/1471-2156-10-15)
Supplement: Additional file 1 — qPCR supplemental methods. Additional information on the qPCR methods used in this manuscript. [file 1471-2156-10-15-S1.doc]

**Supplemental Methods**

*qPCR*

CNVs of interest were validated using a region-specific TaqMan assay. An internal positive control gene that is known to have two copies was included in each well to calculate copy number and to confirm that the reaction amplified successfully. PCR was carried out in a total volume of 10µl, containing 20ng DNA, 1x TaqMan Universal PCR Master Mix (Applied Biosystems), region specific primers, control primers, VIC-labeled region specific probe, and 6FAM-labeled control probe. Amplification was carried out in an ABI PRISM 7900HT Sequence Detection System (Applied Biosystems) with an initial 95°C for 10 minutes, followed by 50 cycles of 95°C for 1 minute, 55°C for 1 minute, and 72°C for 1 minute.  Ct values were generated from a pre-established threshold and delta Cts were calculated from the difference of the known copy number control gene and the CNV test region. The delta Ct values were then treated as a quantitative trait and ANOVA was utilized to estimate the effect of the SNP determined CNV status for the region on the delta ct for that region.
