# Supplementary figures and images for: Copy number variation in African Americans
Source: BMC Genet. 2009 Mar 24;10:15. doi: 10.1186/1471-2156-10-15 (PMC2674062; doi:10.1186/1471-2156-10-15)

**Additional file 2: Replication of CNV analysis in a single individual**

A


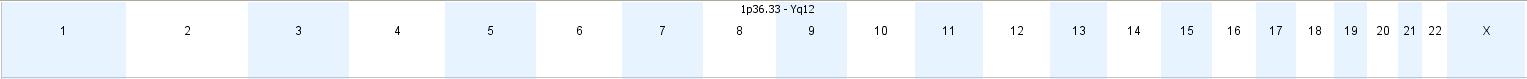


**Chromosome**


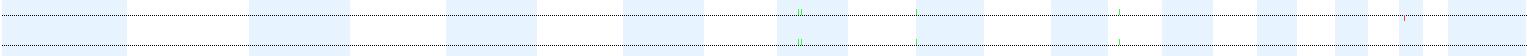


**Ind. 1a**

**Ind. 1b**

B


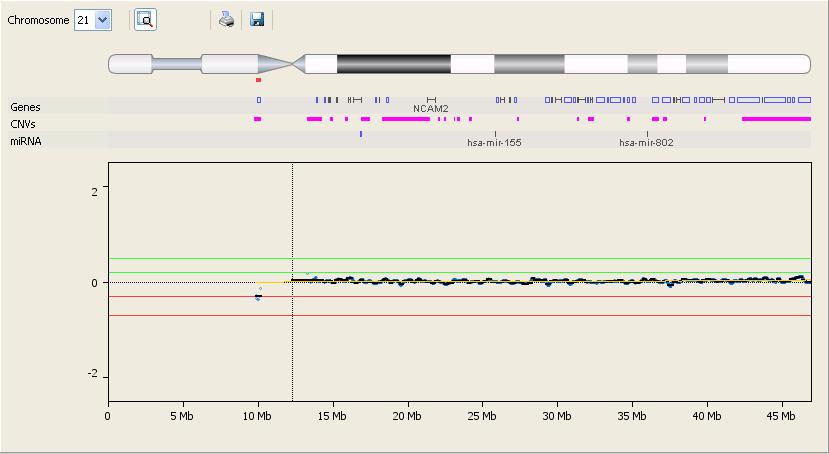


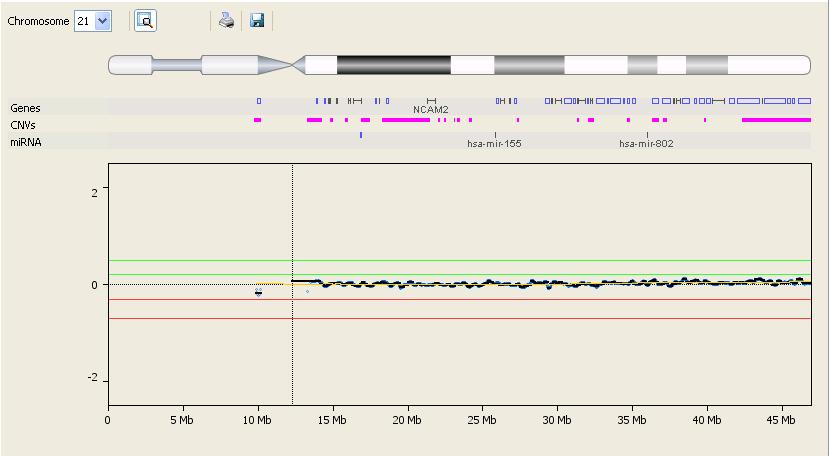

Supplement: Additional file 2 — Replication of CNV analysis in a single individual. A. CNV calls in replicate individual. A green bar above the line for an individual indicates a duplication at that region in the genome, and a red bar below indicates a deletion at that region in the genome. B. Log R ratios for SNPs on chromosome 21. Arrows indicate the region designated as deleted in the first replicate, but not in the second replicate. [file 1471-2156-10-15-S2.doc]
