# Supplementary material for: Copy number variation in African Americans
Source: BMC Genet. 2009 Mar 24;10:15. doi: 10.1186/1471-2156-10-15 (PMC2674062; doi:10.1186/1471-2156-10-15)
Supplement: Additional file 3 — Distribution of the numbers of CNV calls per individual before (A) and after (B) removing outliers. The upper panels of each figure are box and whiskers plots of the data. Each box indicates the interquartile range, each line across the boxes indicates the median, the diamonds indicate the means and 95% confidence intervals, and the whiskers indicate the upper quartile + 1.5 × interquartile range (right side of boxes) and the lower quartile – 1.5 × interquartile range (left side of boxes). [file 1471-2156-10-15-S3.doc]

**Additional file 3: Distribution of the numbers of CNV calls per individual before (A) and after (B) removing**

**outliers**

A.

B.

0

10

10
