# Supplementary material for: Copy number variation in African Americans
Source: BMC Genet. 2009 Mar 24;10:15. doi: 10.1186/1471-2156-10-15 (PMC2674062; doi:10.1186/1471-2156-10-15)
Supplement: Additional file 4 — CNVs identified in more than a single individual. Location, types of CNV events, and within population frequencies of CNVs identified in two or more individuals. Events (relative to reference population): HDel = two copies decrease, Del = one copy decrease, Dup = one copy increase, HDup = two or more copy increase. [file 1471-2156-10-15-S4.doc]

**Additional file 4. CNVs identified in more than a single individual**

| Region | Events | AA Freq. | White Freq. |
| --- | --- | --- | --- |
| chr2:88,922,304-89,912,849 | Del | 0.0078125 | 0 |
| chr5:162,208,673-162,463,912 | Dup | 0.00520833 | 0 |
| chr5:175,325,219-175,711,389 | Dup | 0.00520833 | 0 |
| chr5:175,325,219-175,727,930 | Dup | 0.00520833 | 0 |
| chr7:75,867,431-76,481,102 | Dup | 0.01041667 | 0 |
| chr7:75,929,740-76,481,102 | Dup | 0.03125 | 0 |
| chr7:75,978,391-76,481,102 | Dup | 0.0078125 | 0 |
| chr7:76,017,147-76,481,102 | Dup | 0.00520833 | 0 |
| chr9:114,411,740-114,633,621 | Dup | 0.00520833 | 0 |
| chr10:135,050,636-135,374,737 | Dup,Del | 0.01041667 | 0 |
| chr10:135,133,140-135,374,737 | Dup | 0.00520833 | 0 |
| chr12:31,110,990-31,420,817 | Dup | 0.00520833 | 0 |
| chr12:31,110,990-31,440,999 | Dup | 0.00520833 | 0 |
| chr14:44,759,347-45,130,249 | Del | 0.00520833 | 0 |
| chr14:105,912,153-106,210,891 | Dup | 0.0078125 | 0 |
| chr15:18,427,103-19,325,297 | Dup,Del | 0.0078125 | 0 |
| chr15:18,427,103-19,824,351 | Dup,HDup | 0.00520833 | 0 |
| chr15:18,427,103-19,986,879 | Dup | 0.00520833 | 0 |
| chr15:19,180,021-19,643,166 | Del | 0.0078125 | 0 |
| chr15:19,643,166-20,332,349 | Dup,Del | 0.0078125 | 0 |
| chr15:19,643,166-20,347,279 | Dup,HDup | 0.00520833 | 0 |
| chr17:31,405,303-31,698,335 | Dup | 0.00520833 | 0 |
| chr17:31,415,642-31,698,335 | Dup | 0.00520833 | 0 |
| chr17:31,418,453-31,698,335 | Dup | 0.00520833 | 0 |
| chr22:17,145,634-19,964,004 | Dup | 0.00520833 | 0 |
| chr22:24,070,251-24,240,838 | Dup,Del | 0.00520833 | 0 |
| chr22:24,070,251-24,432,637 | Del | 0.00520833 | 0 |
| chr1:120,599,894-120,992,603 | Dup | 0 | 0.004914 |
| chr1:246,766,039-247,249,719 | Dup | 0 | 0.00737101 |
| chr2:89,089,410-89,912,849 | Del | 0 | 0.01228501 |
| chr2:242,088,986-242,951,149 | Del | 0 | 0.00982801 |
| chr2:242,113,024-242,951,149 | HDel,Del | 0 | 0.01719902 |
| chr2:242,157,542-242,951,149 | Del | 0 | 0.004914 |
| chr3:44,450,530-44,879,083 | Dup | 0 | 0.004914 |
| chr3:44,530,552-44,879,083 | Dup | 0 | 0.004914 |
| chr3:75,506,232-76,039,734 | Dup | 0 | 0.004914 |
| chr3:75,586,858-75,998,876 | Dup | 0 | 0.004914 |
| chr3:75,586,858-76,020,857 | Dup | 0 | 0.004914 |
| chr3:75,662,785-76,020,857 | Dup | 0 | 0.004914 |
| chr4:0-723,505 | Dup | 0 | 0.00737101 |
| chr4:71,599,607-72,057,531 | Dup | 0 | 0.004914 |
| chr4:71,599,607-71,995,478 | Dup | 0 | 0.00737101 |
| chr8:137,779,298-137,892,638 | Del | 0 | 0.004914 |
| chr10:45,438,412-47,711,937 | Dup,HDup | 0 | 0.004914 |
| chr10:45,438,412-47,348,666 | Dup,HDup | 0 | 0.00982801 |
| chr10:45,502,853-46,793,561 | Dup | 0 | 0.004914 |
| chr10:47,067,695-47,348,666 | Dup | 0 | 0.00737101 |
| chr10:47,067,695-47,711,937 | Dup | 0 | 0.00982801 |
| chr12:35,400,000-36,497,952 | Dup | 0 | 0.004914 |
| chr12:35,400,000-36,632,066 | Dup | 0 | 0.004914 |
| chr14:19,272,965-19,594,884 | Dup | 0 | 0.004914 |
| chr15:18,427,103-18,994,147 | Dup | 0 | 0.004914 |
| chr15:18,427,103-19,436,270 | Dup,HDup | 0 | 0.004914 |
| chr15:18,427,103-19,841,337 | Dup,HDup | 0 | 0.004914 |
| chr15:18,427,103-19,199,643 | Dup,Del | 0 | 0.00737101 |
| chr15:18,994,147-19,824,351 | Dup | 0 | 0.004914 |
| chr15:19,171,583-19,854,575 | Dup | 0 | 0.004914 |
| chr15:19,171,583-20,209,311 | Dup,HDup | 0 | 0.01474201 |
| chr15:19,212,555-20,209,311 | Dup | 0 | 0.00982801 |
| chr15:19,361,796-20,209,311 | Dup,Del | 0 | 0.004914 |
| chr15:19,643,166-19,962,117 | Dup,Del | 0 | 0.004914 |
| chr15:19,643,166-20,421,253 | Del | 0 | 0.004914 |
| chr15:19,643,166-19,970,578 | Del | 0 | 0.00737101 |
| chr15:19,841,337-20,209,311 | Dup | 0 | 0.004914 |
| chr16:28,306,730-28,936,772 | Dup | 0 | 0.004914 |
| chr17:41,467,096-41,581,202 | Dup | 0 | 0.004914 |
| chr17:41,469,314-41,932,151 | Dup | 0 | 0.00737101 |
| chr17:41,471,185-41,932,151 | Dup | 0 | 0.004914 |
| chr17:41,471,575-41,932,151 | Dup | 0 | 0.004914 |
| chr17:41,471,909-41,932,151 | Dup | 0 | 0.00737101 |
| chr17:41,472,441-42,150,396 | Dup | 0 | 0.004914 |
| chr17:41,472,441-41,932,151 | Dup | 0 | 0.00982801 |
| chr17:41,473,822-41,932,151 | Dup | 0 | 0.01228501 |
| chr17:41,475,774-41,932,151 | Dup | 0 | 0.01474201 |
| chr17:41,476,064-41,932,151 | Dup | 0 | 0.004914 |
| chr17:41,476,806-41,932,151 | Dup | 0 | 0.00982801 |
| chr17:41,497,758-41,932,151 | Dup | 0 | 0.00982801 |
| chr17:41,500,123-41,932,151 | Dup | 0 | 0.00737101 |
| chr17:41,550,125-41,932,151 | Dup | 0 | 0.004914 |
| chr17:41,562,036-41,932,151 | Dup | 0 | 0.00737101 |
| chr17:41,564,016-41,932,151 | Dup | 0 | 0.004914 |
| chr17:41,564,281-41,932,151 | Dup | 0 | 0.00737101 |
| chr17:41,569,235-41,932,151 | Dup | 0 | 0.01228501 |
| chr17:41,570,077-41,932,151 | Dup | 0 | 0.004914 |
| chr17:41,571,334-41,932,151 | Dup | 0 | 0.00737101 |
| chr17:41,572,051-41,932,151 | Dup | 0 | 0.01228501 |
| chr17:41,573,716-42,147,421 | Dup | 0 | 0.004914 |
| chr17:41,573,716-41,932,151 | Dup | 0 | 0.02948403 |
| chr17:41,581,202-41,932,151 | Dup,HDup | 0 | 0.01474201 |
| chr17:41,587,088-41,932,151 | Dup,HDup | 0 | 0.02457002 |
| chr17:41,591,403-42,150,396 | Dup | 0 | 0.004914 |
| chr17:41,594,804-42,147,421 | Dup | 0 | 0.004914 |
| chr17:41,594,804-41,932,151 | Dup | 0 | 0.01719902 |
| chr17:41,599,921-41,932,151 | Dup | 0 | 0.01228501 |
| chr19:47,964,752-48,536,986 | Dup | 0 | 0.00737101 |
| chrX:51,118,709-51,534,825 | Dup | 0 | 0.004914 |
| chrX:79,946,434-80,414,847 | Dup | 0 | 0.004914 |
| chrX:79,946,434-80,316,347 | Dup | 0 | 0.01474201 |
| chr1:146,740,157-148,060,466 | Dup,HDel,Del | 0.00260417 | 0.004914 |
| chr2:0-217,134 | Del | 0.00260417 | 0.002457 |
| chr2:88,876,198-89,912,849 | Del | 0.01041667 | 0.002457 |
| chr2:89,000,184-89,912,849 | Dup,HDel,Del | 0.02604167 | 0.01719902 |
| chr2:89,220,457-89,912,849 | Del | 0.01041667 | 0.00982801 |
| chr2:91,328,902-91,328,902 | Dup,HDup,Del | 0.11979167 | 0.42260442 |
| chr2:242,138,488-242,951,149 | Del | 0.00260417 | 0.01228501 |
| chr2:242,470,885-242,951,149 | Dup,Del | 0.00260417 | 0.004914 |
| chr7:75,929,740-76,568,388 | Dup | 0.01822917 | 0.004914 |
| chr8:137,793,682-137,890,101 | Del | 0.00260417 | 0.004914 |
| chr9:41,217,099-41,217,099 | Dup,HDup,HDel,Del | 0.4375 | 0.4963145 |
| chr9:43,470,441-45,028,875 | Dup,Del | 0.04166667 | 0.11793612 |
| chr9:46,875,500-46,875,500 | Dup,HDup,HDel,Del | 0.58854167 | 0.71498771 |
| chr9:66,256,186-66,273,146 | Dup,HDup,Del | 0.13541667 | 0.06633907 |
| chr9:67,813,967-69,117,359 | Dup,HDup,Del | 0.10416667 | 0.17199017 |
| chr10:45,949,902-48,022,210 | Dup | 0.00260417 | 0.002457 |
| chr10:45,949,902-47,067,695 | Dup,Del | 0.00520833 | 0.004914 |
| chr10:45,949,902-47,711,937 | Dup | 0.01041667 | 0.01228501 |
| chr10:45,949,902-46,793,561 | Dup,Del | 0.01041667 | 0.01474201 |
| chr10:45,949,902-47,348,666 | Dup,HDup,Del | 0.01822917 | 0.02702703 |
| chr10:46,530,487-47,711,937 | Dup,Del | 0.00260417 | 0.002457 |
| chr10:46,793,561-47,158,916 | Dup | 0.00260417 | 0.002457 |
| chr10:46,793,561-48,022,210 | Dup | 0.00260417 | 0.002457 |
| chr10:46,793,561-47,711,937 | Dup | 0.00260417 | 0.004914 |
| chr10:46,793,561-47,348,666 | Dup,Del | 0.02083333 | 0.002457 |
| chr10:47,067,247-47,711,937 | Dup | 0.00260417 | 0.004914 |
| chr10:134,956,608-135,374,737 | Dup | 0.00520833 | 0.004914 |
| chr10:134,973,380-135,374,737 | Dup | 0.00260417 | 0.002457 |
| chr10:135,005,267-135,374,737 | Dup | 0.00260417 | 0.03194103 |
| chr10:135,064,053-135,374,737 | Dup | 0.00520833 | 0.004914 |
| chr12:31,150,083-31,420,817 | Dup | 0.00260417 | 0.002457 |
| chr12:31,150,083-31,440,999 | Dup | 0.00260417 | 0.002457 |
| chr14:19,272,965-19,471,297 | Dup | 0.00260417 | 0.002457 |
| chr14:19,272,965-19,590,848 | Dup | 0.00260417 | 0.002457 |
| chr14:19,272,965-19,593,328 | Dup | 0.00260417 | 0.002457 |
| chr14:19,272,965-19,494,087 | Dup,HDup | 0.00260417 | 0.004914 |
| chr14:19,272,965-19,489,318 | Dup,HDup | 0.00260417 | 0.01228501 |
| chr14:19,272,965-19,491,207 | Dup | 0.00520833 | 0.00982801 |
| chr14:19,272,965-19,485,438 | Dup | 0.0078125 | 0.01719902 |
| chr14:19,272,965-19,483,260 | Dup,HDup | 0.015625 | 0.01228501 |
| chr14:19,272,965-19,487,782 | Dup,HDup | 0.01822917 | 0.01965602 |
| chr14:19,272,965-19,489,881 | Dup,HDup | 0.02083333 | 0.03685504 |
| chr14:19,272,965-19,489,654 | Dup,HDup | 0.02604167 | 0.04914005 |
| chr14:19,272,965-19,488,714 | Dup,HDup | 0.02864583 | 0.02948403 |
| chr15:18,427,103-20,339,567 | Dup,Del | 0.0078125 | 0.004914 |
| chr15:18,427,103-19,171,583 | Dup,Del | 0.01041667 | 0.02211302 |
| chr15:18,427,103-19,643,166 | Dup,HDup,HDel,Del | 0.08854167 | 0.07371007 |
| chr15:18,427,103-20,209,311 | Dup,HDup,Del | 0.15885417 | 0.15970516 |
| chr15:18,994,147-19,830,911 | Dup | 0.00260417 | 0.002457 |
| chr15:18,994,147-20,209,311 | Dup,HDup | 0.00260417 | 0.02457002 |
| chr15:18,994,147-19,643,166 | Dup,Del | 0.00520833 | 0.00737101 |
| chr15:19,171,583-19,643,166 | Dup,HDup,Del | 0.00520833 | 0.01965602 |
| chr15:19,400,766-20,209,311 | Dup | 0.00260417 | 0.002457 |
| chr15:19,436,270-20,209,311 | Dup | 0.00260417 | 0.004914 |
| chr15:19,643,166-19,968,617 | Dup,Del | 0.00260417 | 0.002457 |
| chr15:19,643,166-20,335,653 | Dup,HDup,Del | 0.00520833 | 0.002457 |
| chr15:19,643,166-20,339,567 | Dup,Del | 0.00520833 | 0.01474201 |
| chr15:19,643,166-20,382,857 | Dup,Del | 0.01041667 | 0.002457 |
| chr15:19,643,166-20,209,311 | Dup,HDup,Del | 0.12239583 | 0.06633907 |
| chr15:19,838,056-20,209,311 | Dup,Del | 0.00260417 | 0.002457 |
| chr15:19,847,297-20,209,311 | Dup,Del | 0.00260417 | 0.00737101 |
| chr15:19,854,575-20,209,311 | Dup,Del | 0.00260417 | 0.004914 |
| chr16:28,306,730-28,870,576 | Dup | 0.00260417 | 0.004914 |
| chr17:41,465,867-41,932,151 | Dup | 0.00260417 | 0.00737101 |
| chr17:41,475,279-41,932,151 | Dup | 0.00260417 | 0.004914 |
| chr17:41,588,328-41,932,151 | Dup | 0.00260417 | 0.02211302 |
| chr17:41,589,928-41,932,151 | Dup | 0.00260417 | 0.01228501 |
| chr17:41,591,403-41,932,151 | Dup,HDup | 0.00260417 | 0.03194103 |
| chr17:41,592,674-41,932,151 | Dup | 0.0078125 | 0.01965602 |
| chr17:41,597,102-41,932,151 | Dup | 0.00260417 | 0.004914 |
| chr17:41,602,588-41,932,151 | Dup | 0.00260417 | 0.002457 |
| chr20:28,106,854-28,143,658 | Dup,Del | 0.06770833 | 0.05651106 |
| chr21:9,887,804-10,189,119 | Dup,Del | 0.06510417 | 0.05159705 |
| chr22:24,070,251-24,236,113 | Dup | 0.00260417 | 0.002457 |
| chr22:24,082,425-24,236,638 | Dup,Del | 0.00260417 | 0.002457 |
| chrX:0-439,193 | Dup | 0.00260417 | 0.002457 |
