# Supplementary material for: Copy number variation in African Americans
Source: BMC Genet. 2009 Mar 24;10:15. doi: 10.1186/1471-2156-10-15 (PMC2674062; doi:10.1186/1471-2156-10-15)
Supplement: Additional file 5 — qPCR of chr. 14 replicates (red cell line, blue whole blood). Bar chart of delta Cts for ten individuals with both whole blood and cell line derived DNAs for the chromosome 14q11 cell line associated CNV. Red bars are cell line DNAs, blue bars are whole blood DNAs. In every individual, the cell line DNAs have a lower delta Ct. Primer sequence for chromosome 14 qPCR: forward-5' CAC TGG CAT TTG GTA TCG T 3', reverse-5' CCC AAA GTG AAA CGT ATT 3'. [file 1471-2156-10-15-S5.doc]

**Additional file 5: qPCR of chr. 14 replicates (red cell line, blue whole blood)**
